# Microbial Symbiosis in Lepidoptera: Analyzing the Gut Microbiota for Sustainable Pest Management

**DOI:** 10.3390/biology14080937

**Published:** 2025-07-25

**Authors:** Abdul Basit, Inzamam Ul Haq, Moazam Hyder, Muhammad Humza, Muhammad Younas, Muhammad Rehan Akhtar, Muhammad Adeel Ghafar, Tong-Xian Liu, Youming Hou

**Affiliations:** 1 State Key Laboratory of Ecological Pest Control for Fujian and Taiwan Crops, Key Laboratory of Biopesticides and Chemical Biology, MOE, College of Plant Protection, Fujian Agriculture and Forestry University, Fuzhou 350002, China; malikbasituaf@gmail.com (A.B.); 000b370305@fafu.edu.cn (I.U.H.); sahito.2k10pt192@hotmail.com (M.H.); 2221903005@fafu.edu.cn (M.A.G.); 2 State Key Laboratory of Wheat Improvement, Shandong Provincial Key Laboratory of Agricultural Microbiology, College of Plant Protection, Shandong Agricultural University, Tai’an 271018, China; muhammadhumza1992@outlook.com; 3 Vector-Borne Virus Research Center, State Key Laboratory of Agricultural and Forestry Biosecurity, Fujian Agriculture and Forestry University, Fuzhou 350002, China; younasali643@gmail.com; 4 State Key Laboratory of Ecological Pest Control for Fujian and Taiwan Crops, Institute of Applied Ecology, Fujian Agriculture and Forestry University, Fuzhou 350002, China; m.rehan@fafu.edu.cn; 5 Guizhou Provincial Key Laboratory for Agricultural Pest Management of the Mountainous Region, Institute of Entomology, and Institute of Plant Health and Medicine, Guizhou University, Guiyang 550025, China; 6 Institute of Plant Health and Medicine, Guizhou University, Guiyang 550025, China

**Keywords:** gut microbiota, insect–plant interactions, microbiome pest management, endosymbionts

## Abstract

Recent advances in microbiome studies have deepened our understanding of endosymbionts and gut-associated microbiota in host biology. Of those, lepidopteran systems in particular harbor a complex and diverse microbiome with various microbial taxa that are stable and transmitted between larval and adult stages, and others that are transient and context-dependent. We highlight key microorganisms—including *Bacillus*, *Lactobacillus*, *Escherichia coli*, *Pseudomonas*, *Rhizobium*, *Fusarium*, *Aspergillus*, *Saccharomyces*, *Bifidobacterium*, and *Wolbachia*—that play critical roles in microbial ecology, biotechnology, and microbiome studies. The fitness implications of these microbial communities can be variable; some microbes improve host performance, while others neither positively nor negatively impact host fitness, or their impact is undetectable. This review examines the central position played by the gut microbiota in interactions of insects with plants, highlighting the functions of the microbiota in the manipulation of the behavior of herbivorous pests, modulating plant physiology, and regulating higher trophic levels in natural food webs. It also bridges microbiome ecology and applied pest management, emphasizing *S. frugiperda* as a model for symbiont-based intervention. As gut microbiota are central to the life history of herbivorous pests, we consider how these interactions can be exploited to drive the development of new, environmentally sound biocontrol strategies. Novel biotechnological strategies, including symbiont-based RNA interference (RNAi) and paratransgenesis, represent promising but still immature technologies with major obstacles to overcome in their practical application. However, microbiota-mediated pest control is an attractive strategy to move towards sustainable agriculture. Significantly, the gut microbiota of *S. frugiperda* is essential for *S. frugiperda* to adapt to a wide spectrum of host plants and different ecological niches. Studies have revealed that the microbiome of *S. frugiperda* has a close positive relationship with the fitness and susceptibility to entomopathogenic fungi; therefore, targeting the *S. frugiperda* microbiome may have good potential for innovative biocontrol strategies in the future.

## Simple Summary

The gut bacterial commensals of Lepidoptera are highly diverse and complex and contain both endosymbionts and transient diet-associated bacteria. Microbiome composition is influenced by (but not limited to) diet, environment, host phylogeny, and ontogeny. Some species of gut bacteria modulate diverse physiological activities, such as host nutrition, detoxification, and immune defense. In addition, they have an impact on insect–plant interaction by manipulating plant host preference, overcoming feeding limitations, and, possibly, inducing dysbiosis. Moreover, these microbiotas emit volatile organic compounds, which may attract or repel natural enemies of the host insect. For example, the gut microbiota of *Spodoptera frugiperda* is crucial for its ability to adapt to diverse host plants and different environments. This is a promising route for the discovery of new biocontrol strategies. This review highlights gut microbiota as one of the most promising targets in the development of novel strategies for pest control, such as symbiont-mediated RNA interference and paratransgenesis. However, significant hurdles remain in deciphering the fundamental mechanisms of insect–microbe–plant interaction, and in the practical translation of laboratory findings to the field.

## 1. Introduction

The widespread symbiotic relationships that are observed between insects and microorganisms have furthered their application in explaining community and physiological processes [1,2,3]. Butterflies and moths, or the Lepidoptera, are the second largest order of insects, which is not just represented by some of the most crucial pollinators but also by important crop pests. This clade represents one of the most complex and widespread symbiotic associations reported for animals [4,5,6]. Recent applications of high-throughput technologies have uncovered the unparalleled diversity of microorganisms as well as their transmission and effects on Lepidoptera [7,8,9]. The plant–fungal microbiota interactions in this context show a continuum of interactions that vary from pathogenic to mutualistic.

Studies on the Lepidoptera microbiome early focused primarily on insect–pathogen interactions. Nevertheless, recent evidence supports that symbiotic microbes modulate host interactions. Although many reviews have addressed pathogens [10,11,12], the present synthesis concentrates on nonpathogenic microorganisms. The focus of the review is the variety and abundance of microbes in Lepidoptera. It starts with intracellular endosymbionts, continues with extracellular microorganisms, in which ectosymbionts in the gut are well represented. Microbiome surveys using sequencing-based technologies have been performed across diverse lepidopterans (>100, as of date). However, the functional roles of the majority of these microbiomes are poorly understood.

**Figure 1:**
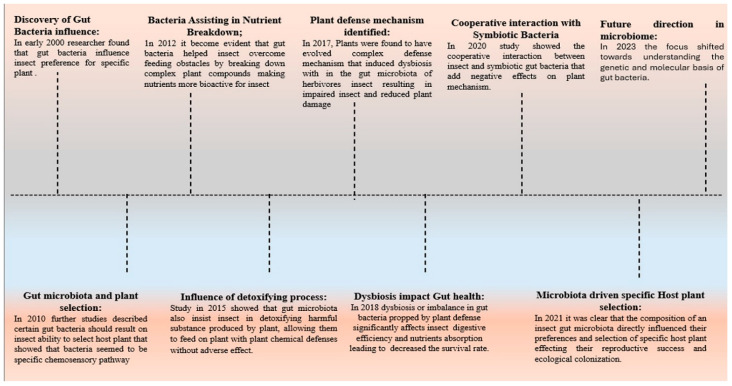
Schematic illustration of the gut microbiota of lepidopteran insects. The figure depicts the different microbe communities: intracellular endosymbionts and extracellular ectosymbionts of an insect gut. It portrays the relationship of these microbes with the host, and focuses on functions, such as nutrient provisioning, digestion, detoxification, and insect behavior modulation. The diagram also indicates the dynamics of microbial transmission, the impact of environmental and dietary conditions on microbiota, and the putative functions of these microbes in insect–plant interactions.

The insect gut microbiota is rich and diverse in its composition [10]. That specific gut bacteria are involved in the adaptation of insect pests to their host plants has been well-documented, including the provision of essential nutrients, digestion, detoxification, and insect behavior [13,14,15]. These mutualistic associations between insects and their gut microbiota, termed the ‘social life of insects’ by Brune (2014) [16], underpin many evolutionary scenarios and diversification events. However, these symbioses are problematic if you think about pest control. A number of reviews indicate that, under certain circumstances, including the presence of a third species in pest–plant interactions [17] or in response to certain environmental stress [11,18], these helpful gut bacteria can act as antagonists and indirectly harm their insect hosts. Therefore, the gut bacteria and their roles in complex multispecies interactions are promising targets for biocontrol and biopesticides. While there are extensive reviews on the functions of gut bacteria in insects [19,20], less attention has been given to their various roles in insect–plant interactions through the lens of a cascading multispecies interaction model [20,21,22]. A simplified diagram of gut microbial biota is presented in Figure 1. The Incompatible Insect Technique (IIT), a promising Wolbachia-based approach, has demonstrated significant potential in controlling vector populations and other insect pests. By releasing male insects infected with a specific Wolbachia strain to mate with wild females, which may be uninfected or harbor a different strain of Wolbachia, the resulting offspring fail to develop or die due to reproductive interference. This mechanism leads to a gradual reduction in target pest populations and has been particularly effective in controlling mosquito vectors of diseases such as dengue, Zika, and malaria, as well as agricultural pests [6,13]. IIT offers a sustainable alternative to chemical control methods by relying on naturally occurring Wolbachia and avoiding the introduction of genetically modified organisms into the environment. Despite its promising results, further research is needed to optimize release strategies, assess long-term efficacy, and ensure ecological safety, making IIT a powerful yet evolving tool for integrated pest management and vector control [20].

In this review, we discuss the key role that gut bacteria play in lepidopteran systems and how they affect insect–plant interactions, the biology of the host and the management of pests, and how such mechanisms could be exploited in agriculture. The objective is to review existing knowledge, summarize the most important microbial taxa, and critically assess the effects of microorganisms on their lepidopteran host. This review synthesizes the current literature and discusses the new areas of opportunity for harnessing insect–microbe interactions in improving sustainable pest management, biotechnology, and the regulation of insect–plant associations.

## 2. Microbiome Diversity in Lepidoptera

Lepidoptera, during their holometabolous life cycle, have access to different dietary niches and substrate sources. Association with microorganisms continues in both larval and adult stages, which may be either endosymbionts in the host or also ectosymbionts on the body of the host and the gut. Although the number of endosymbiont taxa of a given host is usually low, the number of ectosymbionts is abundant. The host microbiome is a dynamic ecosystem that is influenced by different factors such as host-related factors and environmental factors. The environment is known to be an important source of microorganisms and has a significant influence on the evolution of insect–microbe associations. There are two pathways for microbial transfer: horizontally, from one generation to the next, or vertically, across generations. Here, we consolidate taxonomic contents and structural patterns of Lepidoptera microbiomes, including bacteria, fungi, and viruses, in this review. We further discuss the environmental and host-associated determinants shaping the composition of the gut microbiome, as shown in Table 1.

## 3. Endosymbionts

**Figure 2:**
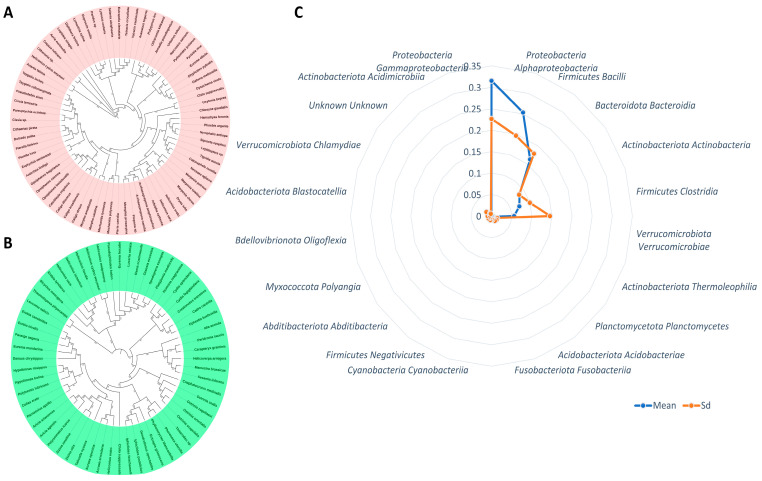
Host phylogeny COI-based and gut microbial abundance across insect–plant system (**A**,**B**). Maximum-likelihood phylogeny of insect hosts with radar plot shows microbial taxon abundance (**C**). The blue-colored line indicates mean, and the red-colored line denotes standard deviation (Sd) of the co-abundance profile of the microbiome in the gut of lepidopterans.

Endosymbiosis with bacteria occurs widely in insects, and the most common bacteria are *Wolbachia Alphaproteobacteria*, *Rickettsia Alphaproteobacteria*, and *Cardinium* [33]. A similar relationship of prevalence of endosymbionts is observed in Lepidoptera as well (Figure 2A). However, compared to *Wolbachia*, a limited number of studies have been reported in Lepidopteran endosymbionts [34]. A study of around 300 Lepidoptera species showed that some 80% of them are infected with *Wolbachia* [31]. This ancient symbiont was likely acquired by Lepidoptera between 22.6 and 4.7 million years ago [27].

Wolbachia is highly diverse both biologically and in terms of host usage; there are a reported 16 supergroups that fall within *Wolbachia pipientis* [35]. In Lepidoptera, *Wolbachia* strains are mainly represented by supergroups A and B, with supergroup B being more commonly detected in this insect order [27]. Although Wolbachia is predominantly vertically transmitted from mothers to offspring, there are well-documented examples of horizontal transmission, which probably occur via direct exchanges of bacteria or resources along with shared food resources or interactions with natural enemies like parasitoid wasps [27]. This bimodal transmission mode underscores the intricate relationship of *Wolbachia* with its Lepidopteran hosts, with ecological and evolutionary implications.

Among Lepidopteran endosymbiotic bacteria, *Spiroplasma* is the second most common and is present in 4–7% of the surveyed engineering species and is notably prominent in Danaus butterflies (Figure 2A). In contrast to Wolbachia [15], most *Spiroplasma symbionts* are commensal and have been less well investigated. Despite being a cosmopolitan pathogen, only one species of Rickettsia has been identified in Lepidoptera [19]. Since *R. felis* infects a wide range of hosts, additional studies will be necessary to ascertain whether its association with Lepidoptera is a specific relationship, possibly with disease implications, as opposed to a coincidental one.

Recent metagenomic analyses have described frequent interactions between *Arsenophonus* and Lepidoptera [29] (Figure 2A), but their distribution among all lepidopteran super-families is yet to be elucidated completely. To our knowledge, the presence of Cardinium has not been reported in Lepidoptera. Also, other very common endosymbiotic bacteria, such as *Hamiltonella*, have not been found in this group; if present, their incidence is likely very low.

Fungal endosymbionts of Lepidoptera are less common than in other orders of insects [36]. However, a single exception is the very internal yeast-like endosymbiont *Purpureocillium* sp. (Ascomycota) isolated from the Thitarodes moth, which presumably results in its maternal transmission through female gonads to progeny [37]. Recent developments in metagenomic sequencing approaches have improved our understanding of nonpathogenic viral endosymbionts of insects. These vertically transmitted viral symbionts can sweep rapidly through host populations, frequently having complex and, at times, counterintuitive effects on their hosts [38]. For instance, sigma-like viruses (negative-strand RNA viruses) are present in the nymphalid butterfly *Pararge aegeria*, infecting on average 74%. The significance of these cocirculating viruses in the host is not clear and needs further study [39]. Furthermore, a novel double-strand RNA virus infecting female *Homona magnanima* (legume pod borer) is also maternally transmissible, with asymptomatic infections in females. In contrast, male WSB larvae die from the virus in late larval development [40]. A common densovirus (ssDNA virus) has also been discovered in wild populations of *H. armigera* with an infection rate of over 67%. The virus seems to have a beneficial effect on its host without the specific mechanisms having been worked out yet, which is an interesting area for future research [41].

## 4. Gut Microbiome Dynamics

Ectosymbiotic microorganisms have shown their ability to be sustained and maintained across the grasshopper tissues, like the cuticle, hemolymph, and gut epithelium. Although ectosymbionts have been better studied in relation to their digestive function [42], the digestive tract of larval Lepidoptera is generally fairly simple, and is not highly specialized either functionally or compositionally, occupying a significant portion of the body cavity. Unfortunately, metamorphosis brings about profound changes in morphology and the chemical environment of the digestive system, many of which are reflected in alterations of the host diet. Herbivory is a common lifestyle among the insect order Lepidoptera, whose larvae primarily consume leaves, but also flowers; some are (secondary) root feeders, or (in a few microlepidoptera families) leaf miners. Adult Lepidoptera consume nectar or fluids from rotting fruit/tree sap. The microbial communities in Lepidoptera have been mainly studied through bacteria, but not so much for other microbes in the order Lepidoptera (Figure 2B). This trend highlights the importance of further research into microbial diversity in Lepidoptera.

In the last decade, most studies that have focused on the gut microbiota of invertebrates were carried out in larval Lepidoptera, where the gut acts as a very unfriendly environment for microbial proliferation. Factors include high alkalinity, which leads to rapid movement of food, continuous renewal of the peritrophic matrix, and antimicrobial peptides of host origin. Nevertheless, despite the extreme conditions met in the gut of the larvae, microbial populations in the larval gut have been estimated, using classic culture methods and direct gut tissue analysis, to be in the order of 10^7^–10^13^ microorganisms per single larva [23,43,44]. Frass (insect feces) studies, however, report much less bacteria, ~104 16S rRNA copies/g, of which most come from the diet and not the gut microbiota [39]. Nevertheless, recent studies have revealed that the abundance of bacteria is still relatively low in adult Lepidoptera, but possibly the amount is substantial compared to what was believed before. Adult gut microbiomes, for example, have been estimated to include anywhere from 5 × 10^5^ to 1 × 10^11^ 16S rRNA copies per butterfly, with a median of 7.5 × 10^8^ [6]. This underscores an intriguing aspect of microbial colonization, even at the adult stages, and raises questions for future studies of microbial roles throughout Lepidoptera life history.

Use of sterile diets in laboratory experiments, combined with controlled inoculation of specific microbes, has shown that some microbes are indeed able to take up residence in, and can proliferate within the Lepidopteran gut [21,45]. These microorganisms are found in dense aggregates in microscopic sections [46,47], suggesting that they attach to the gut lining and become incorporated into the peritrophic matrix, as shown in Figure 2C. This initial stage of contact with the host and the subsequent formation of a biofilm is a pivotal stage for the ontogeny and evolution of these microbial consortia. Within the wild larval gut, microbial communities are highly variable, depending on local environmental conditions, but frequently very stable over time and space [48,49]. Interestingly, a set of bacterial taxa is harbored by particular Lepidoptera species independent of differences in their diets, indicative of a unique host-specific microbial signature [14,50,51,52,53,54,55,56,57,58,59]. Conversely, possibly only a minority of their microbiome will be comprised of transient microbial associates in other species, thus emphasizing the complexity and differences in microbial community dynamics in Lepidoptera. This variation, as well as specificity, opens exciting opportunities for dissecting functional contributions of these microbial communities to insect biology.

These variances highlight the intricate and dynamic interaction between Lepidoptera and their gut microbiome. Due to interactions within digestive systems, microbial colonization in Lepidoptera is assumed to be a non-random process. Microbiota assembly in insects takes place through various routes, such as food, water, and soil. For instance, *Tyria jacobaeae* larva can transmit certain microbiota to its generations [48]. Flowers can also act as a secondary source of adult Lepidoptera-specific gut microbiota [60], suggesting an environmental transmission route as well as host-specific interactions. These findings are also consistent with the observation of a strict specificity of gut microbiomes in other plant-feeding insects (e.g., heteropterans) [60,61], but including an environmental transmission part. In addition, sociality within some species also enhances the exchange of microbes between hosts. For instance, shared feces and feeding sites in gregarious larvae may enable host-to-host microbiota transmission. The acquisition of new facultative symbionts, in turn, could contribute to the host’s adaptability relative to environmental or physiological challenges [62].

Genomes of extracellular gut symbionts and food-associated bacteria are particularly flexible, being highly prone to gain or loss of accessory functional genes, which is a hallmark for many free-living bacterial groups [63]. Some of these symbionts can progress into tightly integrated endosymbionts, which could lead to extremely specialized symbiotic associations within their hosts [64]. This evolution may result in the acquisition of new functions for the host [65]. Yet, use of environmental transmission for microbial contributions to host fitness could undermine the long-term stability of these associations.

Transmission of resident microbiota from parents to offspring vertically has been observed in some cases [18,28,57,60,66,67]. For instance, green fluorescent protein (GFP)-labeled Enterococcus was detected in every life stage, including generations, of Spodoptera littoralis individuals following ingestion [68]. Further, microbes often associate with eggs, as hatchling larvae eat their eggshells and with them the associated microbes. So, in this way, the microbes can colonize and multiply in the host [18]. Horizontal transmission within the larvae of the same species, including the transovarial, where some bacteria located in the larval gut are able to cross the gut epithelium and enter the hemocoel, and finally the eggs [66] during the development within the *Galleria mellonella*. These bacteria are concentrated in the ovaries and occur predominantly in the eggshell (chorion) of developing eggs.

Lepidopteran enteric microbiome has a varying proportion of transient, opportunistic, and resident symbionts. Commonalities in microbiota structure and composition result from a combination of ecological factors and transmission pathways. However, our understanding of gut symbionts in Lepidoptera is still minimal, especially regarding their localization, transmission routes, and the interplay between vertical and horizontal transmission. To comprehensively assess the Lepidopteran gut microbiome, a more fine-scale investigation should be performed, examining the roles of the resident versus the transitory microbes, driving forces of population and community alterations, a priority effect of assembly, and also the intricate microbiota interactions, such as competition, within the host. This enhanced knowledge will be invaluable in understanding the ecological and evolutionary processes that shape these microbial communities.

## 5. Key Factors Influencing the Composition and Function of the Insect Gut Microbiome

Even though core microbiome markers have been identified for some Lepidoptera lineages, the community composition of the gut microbiome can vary substantially between individuals of the same species [6], which is known to be the case for other animal groups. This appears to be the case to a greater extent for larvae than adults, even if some taxa also occur across life stages [26,68,69]. Microbiome composition may deviate in response to multiple proximate (diet, habitat) and ultimate factors (phylogeny, ontogeny) [70] (Table 2).

### 5.1. Diet and Environmental Influences on the Gut Microbiome in Lepidoptera

Lepidoptera mainly obtain gut microbes by feeding, due to the few food sources that are pathogen-free, and this promotes the interactions between transient and resident microbiota. Some species ingest eggshells at the time of eclosion, and these can be potential sources for more diverse microbial communities [7,71]. However, much less is known about the interactions between species in the gut, such as how foraging larvae compare foraging on plants with the established microbiota. Resident microbes may be swapped out for newly encountered beneficial microbes during feeding, whereas other taxa may be ejected. Adults drink different sugar solutions, as nectar, that may introduce further microbial communities. However, it is yet to be determined if nectar-associated microbiota is able to establish and maintain in the gut community. Intuitively, a more expansive study showed that diet guilds accounted for a mere 23% of the variance in gut microbiomes [6], indicating that factors other than diet contribute significantly to the influence on the Lepidoptera gut microbiome.

Beyond nutrition, the host microhabitat significantly influences microbiome diversity. Plants harbor a diversity of ecological niches where microbial communities are modulated by environmental conditions and spatio-temporal dynamics. These plant niches, in turn, influence the herbivorous insects that feed on them [56]. Furthermore, a number of Lepidoptera species acquire soil-dependent gut microbiomes. For example, cabbage moths (*Mamestra brassicae*) have been shown to obtain almost their whole gut microbiome from the soil, rather than from their host plants [49]. This illustrates the intricate nature of microbial procurement in Lepidoptera and underscores the different ecological influences that construct their gut microbiomes.

### 5.2. Influence of Host Phylogeny

The host phylogeny is a key factor determining the relationships with microbial taxa. For example, three Lepidoptera species that were all fed on the same host plant and kept in the same environment differed markedly in their gut microbiomes [19]. In a similar vein, host species contribute around a quarter to a third of the variation in gut microbiomes in butterflies [72], reinforcing the importance of host-specific factors, such as physiology and behavior that are conserved across phylogenetic lineages, in determining microbial community composition. The physiology of the gut has been known to be a major determinant of microbiome diversity in vertebrates [73], and a similar process likely underlies microbiome dynamics in invertebrates. Moreover, phylogenetically related behavior features may serve as intermediaries in the association between microorganisms. Species might also be exposed to different communities of microorganisms as a result of habitat preference; for example, forest-dwelling Lepidoptera are more likely to encounter soil- and wood-decay fungi than their canopy-dwelling relatives [6]. In addition, the daily activities of flies (i.e., feeding and movement) also may impact access to different microbial resources, which may drive variation in microbiome composition across different ecological roles [70]. This emphasizes the intricate interaction of host phylogeny, behavior, and microbial exposure in shaping gut microbiome structure.

### 5.3. Impact of Developmental Transitions on Microbiome Composition

A comparison of microbiota diversity throughout Lepidopteran ontogeny demonstrates that bacterial diversity in larval gut microbiomes is flexible, with substantial changes among instars [22]. Older-instar larvae are mostly studied in field surveys, and this may lead to biases when interpreting microbial functions and their ecological importance. The microbiome undergoes a dramatic transition from larva to adult. Larvae eject their intestines of the last instar with their actual contents just before they pupate, which in turn could promote a deep reorganization of the microbiome at the metamorphosis, involved in the colonization of new environmental niches. Although bacteria acquired post-emergence tend to be different from pre-pupal taxa, some microbial transmission is evident over the larval-adult transition, with the presence of particular bacteria continuing through the pupal stage in some instances [74]. This complexity of bacterial dynamics during Lepidopteran development underscores the necessity for a more thorough investigation of shifts within the microbiome at the time of metamorphosis.

### 5.4. Captivity and Rearing Practices: Unveiling Their Impact on Microbiome Dynamics

As with other insects, the gut microbiota of Lepidoptera reared in laboratory settings is qualitatively and quantitatively different from that found in field-collected ones [69]. Whereas wild populations can hold a wide range of microbiota, those raised in the laboratory generally only carry a simplified microbial community consisting primarily of one or a few species [53]. Although there are shared bacterial taxa in health vs. disease or disease-free vs. diseased states, laboratory microbiomes are often low in diversity as compared to their wild counterpart, which makes it challenging to interpret the ecological and physiological relevance.

Studies of the microbiomes of Lepidoptera demonstrate considerable species-level variation but also conservation at the phylum level. The source and content of the gut microbiota are probably influenced by factors including diet, habitat, and environmental microbial reservoirs. For subsequent investigations, we need to differentiate transient and resident microbes and clarify their functions [75]. In addition to the role of the innate mechanisms described here, the plasticity of gut microbiota in Lepidoptera could be enhanced by adaptive immune mechanisms, which might bring about temporal environmental adaptability or offer (redundant) functional benefits.

### 5.5. Unraveling Symbiotic Alliances: Lepidoptera and Their Gut Microbiota

In Lepidoptera, the gut microbiome also makes an essential contribution to host nutrition and protection, through enhancement of enzymatic potential and secretion of gut enzymes, which contribute to the degradation of complex compounds and detoxification of some toxicants. Symbionts belonging to phyla Proteobacteria and Firmicutes produce cell-wall degrading enzymes (plant degradation enzymes), cellulases, hemicellulases, and pectinases, contributing to the digestion of plant material. For example, the gastrodermal bacteria from *Anticarsia gemmatalis* can secrete trypsins to help digest the protein [76] and the predominant fungi in the larva of Thitarodes can secrete amylases, lipases, and proteases to process the nutrients [37].

With adult Lepidoptera, the gut microbiomes are specialized to metabolize diet-specific compounds. Gut symbionts in *P. xylostella* synthesize essential amino acids such as histidine and threonine that aid in providing nutrition to the host [77]. Curiously, in certain Tortricidae, the plant pathogen *Botrytis cinerea* appears to be a mutualist producing sterols that can affect the host’s life history [30]. The microbiome also promotes host development, as observed in *Pieris canidia*, where it enhances host survival and body weight [78].

In addition, the gut microbiome is essential for the detoxification of plant toxins and xenobiotics. Acinetobacter is capable of catabolizing phenolic compounds [63], and stimulates growth in Lymantria dispar on phenolic-rich diets [79]. Furthermore, *S. frugiperda* is advantaged by Enterococcus and Enterobacter isolates that enable this species to process an otherwise nondigestible diet, thereby increasing its survival and performance [80]. Detoxification mechanisms—for example, taxon-specific gene clusters identified in the gut metagenomes of Spodoptera species and *P. xylostella*—further support the significance of microbiomes in resistance [77,80]. Enrichment of Enterococcus in *S. littoralis* has been associated with the stimulation of methomyl insect tissue tolerance by the induction of metabolic levels. These results highlight that the microbiota significantly contributes to insecticide degradation and open a way for biocontrol applications (Table 3).

Gut microbiomes are important in maintaining immunity and defending against pathogens in Lepidoptera. For example, Enterococcus mundtii in the larval midgut of *Spodoptera littoralis* secretes strong and stable broad-spectrum antimicrobial peptides that selectively kill *Bacteroides* spp., eradicating the pathobionts in the gut and creating favorable conditions for a healthy gut microbiota and reduced infection risk [81]. Nonetheless, some lepidoptera species appear to form intimate relationships with enterococcus, which includes a highly heterogeneous group of bacteria that can act either as mutualistic symbionts or as pathogens, depending on the situation [5].

**Figure 3:**
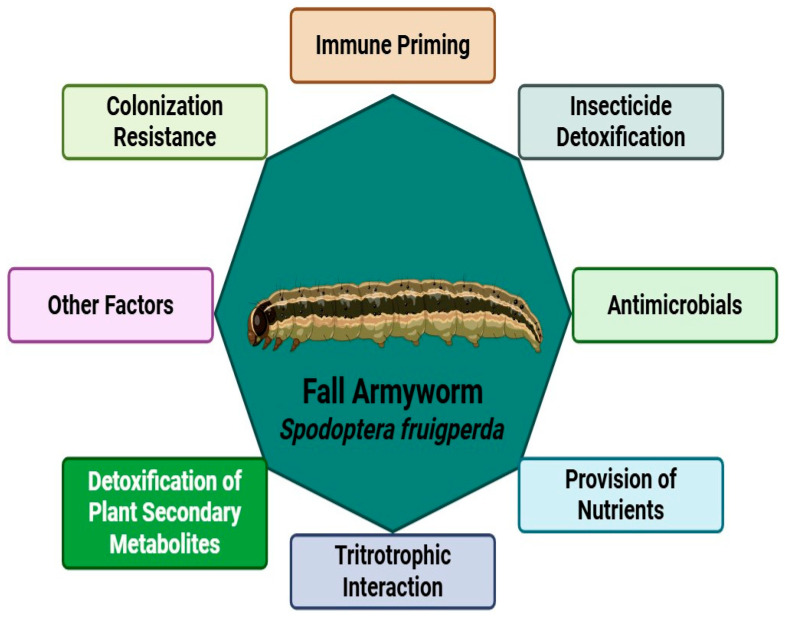
Functional diversity of the Lepidoptera microbiome. Multifaceted strategies employed by Fall Armyworm (*S. frugiperda*) to thrive in its environment, including defense mechanisms, metabolic adaptations, and ecological interactions.

The insect cuticle is also colonized by microbial communities that play a role in host defense. Microbial communities on Citrus butterfly (*Pieris* spp.) wings exhibit potential antagonistic effects. Microbial assemblages in the Citrus butterfly, which are short-lived on the wings, presented antibacterial, antifungal, and biosurfactant activities, including against *Streptomyces* sp. A number of these microorganisms are situated on the cuticle prior to coming into direct contact with environmental media, and as such are involved in protecting the host [82]. Antimicrobial-based searches of insect symbionts have yielded a wealth of bioactive molecules, in particular peptides and polyketides [47]. Taken together, these data emphasize the multifunction of Lepidoptera microbiota that involves detoxifications, immune responses, and, potentially, improved stress tolerance, as well as the crosstalk among detoxifications, Lepidoptera–host immunity, and gut microbiota, as shown in Figure 3.

## 6. Gut Bacteria as Key Modulators of Insect–Plant Interactions

**Figure 4:**
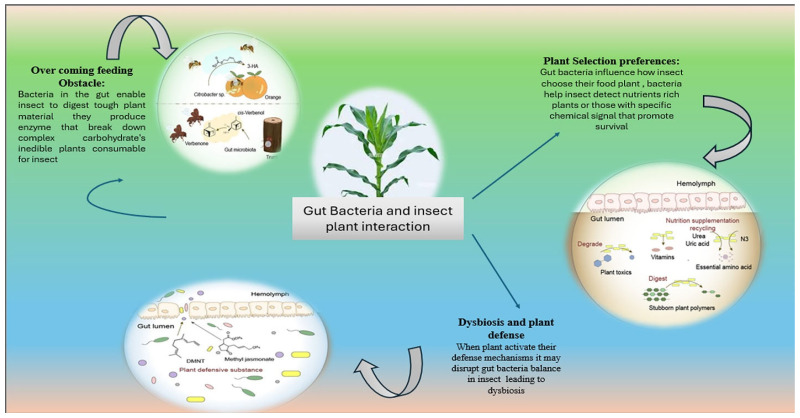
Roles of gut bacteria in influencing insect behavior and physiology. Gut bacteria mediate plant selection preferences in insects, aiding in the identification of suitable host plants. Gut bacteria help insects overcome feeding challenges such as low nutrient availability, indigestible plant tissues, and plant toxins. Dysbiosis in the gut microbiota caused by plant defenses or inadequate nutrition can be detrimental or lethal to insects.

Herbivorous insects need to successfully make use of the resource mosaic of plants, which play a major role in the lives of these animals [83], and have evolved specific mechanisms to that effect. Similarly, these adaptations are probably influenced by direct and indirect associations between the insect gut microbiota and the host plants [84]. Of these, some gut bacteria have established ecological functions that have a direct impact on insect behavior and physiology. These functions can be classified generally, as per Table 4 and as per the schematic in Figure 4.

**Plants as Selecting Mediator:** The gut microbiome may mediate the plant preference of some herbivorous insects, leading the insects to choose appropriate host plants for survival and reproduction. This latter capacity is important for the insect’s ecological and evolutionary success.

**Overcoming Feeding Restrictions:** After searching and locating their host plants herbivorous insects have to deal with several constraints including low nutrient availability, indigestible tissue, and plant toxins [84]. Some gut bacteria possess enzymatic functions that provide insects with an advantage to bypass these defense barriers to feed, grow, and reproduce on host plants successfully.

**Dysbiosis and Destruction:** Conversely, chemicals or nutrient imbalances can cause the insect to have a misshapen gut flora (dysbiosis). This microbial dysbiosis is frequently harmful to insect physiology and fitness. These continued relationships between herbivores and host plants serve to emphasize the centrality of gut-associated bacteria in mediating herbivore–plant–insect associations in the maintenance of these ecological relationships.

## 7. Unraveling the Role of Gut Microbiota in Shaping Insect Host Plant Selection

Host plants are important to the fitness and offspring performance of herbivorous insects. This selection is largely mediated by the chemosensory systems of insects, which perceive and interpret plant volatile organic compounds (VOCs) to make rational behavioral decisions [85,86], which are modified by microorganisms as well. Fungi, endophytes, and plant pathogens have all been implicated in influencing the selectivity of an insect’s choice of host plant, and some gut bacteria are noteworthy as well [87,88]. For example, *Frankliniella occidentalis* prefers to oviposit on thrips-damaged leaves rather than on freshly emerged leaves, and this preference is connected with thrips gut bacteria that can be spread to the leaf surface [89].

Insects can inoculate host plants with insect gut bacteria during foraging, defecation, or oviposition. Once inside the plant, these bacteria multiply using some of the plant’s nutrients and produce VOCs that change insect behavior, attracting or repelling different insects. For instance, *Citrobacter* sp. from the oriental fruit fly (*Bactrocera dorsalis*) generates 3-hexenyl acetate (3-HA) to call other females to oviposit into the host fruit [90]. The gut microbiome of the red turpentine beetle (*Dendroctonus valens*) also synthesizes verbenone, a multitasking pheromone used to relay its condition to potential colonizing beetles on the pine tree host [91,92].

Volatile compounds emitted by gut microflora not only help insects to choose their behavior, but also reflect symbiotic benefits in such interactions. As insects are seeking egg-laying sites, bacteria in their gut are provided with chances of dispersal and establishment. While relatively few studies to date have pointed to gut bacteria–host plant relationships, the reciprocal advantages to both insects and bacteria indicate that such interactions may be more widespread than was previously shown. It is conceivable that similar mechanisms will be identified in other insect species in the future, revealing complex interdependencies between insects, their gut microbiomes, and host plants.

## 8. Harnessing Insect Gut Microbes: How Symbiotic Bacteria Break Down Plant Polymers and Supply Essential Nutrients:

The relatively low enzymatic capacity of most insects to digest complex plant polymers such as cellulose, pectin, or lignin constitutes a major challenge for them. For these polymers, particular enzymes are also needed, such as cellulase, pectinase, and ligninase, but insects produce very few of these [76]. However, the gut microbiome in insects decides to enhance the insects’ digestive efficiency through enzymatic activity.

Cellulosics, the main component of the plant cell wall, are a significant source of carbon and occur as either crystalline or amorphous cellulose [93]. To be utilized, the cellulose should be hydrolyzed to simpler sugar units by cellulases. In this, it typically depends on gut bacteria. For instance, cellulase-producing microorganisms, Streptomyces, and Pantoea that are found in the gut of the invasive wood-feeding wasp (*Sirex noctilio*), enzymatically hydrolyze cellulose, allowing the host to acquire nutrients [72]. It has been demonstrated that the same cellulase-producing bacteria were found in other insects, such as termites (*Macrotermes gilvus*) [94], beetles (*Lepidiota mansueta*, *Odontotaenius disjunctus*) [95,96], and moths (*Cossus cossus*, *Diatraea saccharalis*) [97,98]. These include cellulose moieties, the cellulase-producing bacteria from the species *Providencia* sp., *Bacillus* spp., and *Klebsiella* spp. with similar enzymatic activity.

Plant cell walls consist mainly of cellulose and hemicellulose fibers embedded in a pectin matrix, which has to be degraded to produce substrates for other enzymatic activity [99]. Pectin is also degraded by microbial activity in the gut that is capable of producing pectinases. For instance, the gut symbiont Stammera in the leaf beetle (*Cassida rubiginosa*) harbors genes related to pectin degradation [100]. Similarly, pollen-reliant insects need to overcome the tough pollen wall. The outer pollen wall is primarily made of sporopollenin, and the inner one is made of pectin [101,102]. The gut bacteria *Gilliamella apicola* of honeybees harbor genes associated with pectin degradation, which were discovered using metagenomic studies, and in vitro assays have verified its pectinase activity [103].

Another complex natural polymer that is less digestible is lignin, one of the polymers that is contained in plant cell walls. Fungi are the main organisms depolymerizing lignin, but some gut bacteria are also involved in this process [104]. Bacterial species that degrade lignin have been found in termite intestines [104,105,106]. Moreover, transcriptomic studies have also confirmed that midgut microorganisms contribute to lignin degradation in the longhorn beetle (*Anoplophora glabripennis*) [107,108]. These results highlight that enteric bacteria can be readily adapted to break down structured plant polymers.

In addition to structural defenses, herbivorous insects have to manage nutritional constraints because plant diets are usually poor in one of the essential nutrients required for survival and growth. Insects have evolved gut microbiota-mediated mechanisms to attenuate these nutritional problems. Nitrogen is often the nutrient that limits in herbivore diets [109]. Furthermore, plants may be inadequate in essential amino acids (EAAs) that are essential for insect growth and development [110]. The bacteria in the gut are crucial for correcting these deficiencies. For example, some bacteria in the gut are known to help fix nitrogen. These beetles and the *Tephritid fly*, the medfly *Ceratitis capitata*, harbor bacteria capable of fixing atmospheric nitrogen into bioavailable (usable) forms [111,112]. Moreover, the gut microbiota is involved in nitrogenous waste recycling (NWR), which is also the reabsorption of certain waste products, such as ammonia or uric acid, as EAAs that can be reabsorbed by the insect. Symbiotic bacteria participate in this process, and symbiotic bacteria such as *Morganella morganii* and *Klebsiella oxytoca* in the oriental fruit fly (*Bactrocera dorsalis*) play major roles [113]. In the same way, the recycling of nitrogenous wastes and the synthesis of EAAs necessary for growth and development in leafcutter ants depend on gut bacteria [114].

Plants conduct materials through a sap derived from the xylem or phloem, and the nutrient content of sap is usually low and variable [115]. As a result, *Drosophila melanogaster* feeding on sap from some plants becomes flatworm deficient. However, gut bacteria make up for some of those gaps by synthesizing vitamins for their hosts. For example, in the bean bug *Riptortus pedestris*, symbiotic bacteria synthesize necessary B vitamins, which are not found in the soybean-exclusive diet of the insect [116]. The genomic profiles of the gut bacteria in herbivorous ants (*Dolichoderus* sp.) show genes that encode pathways for the production of multiple vitamins and EAAs [117]. Different strains of bacteria can have differing levels of ability to produce some vitamins. For example, the *thiE* gene for vitamin B1 biosynthesis is present in the majority of members of the Bartonellaceae family, and only a single strain contains all genes required for the de novo synthesis of vitamin B3.

## 9. How Gut Bacteria Modulate Third-Trophic-Level Dynamics to Influence Insect–Plant Relationships?

**Figure 5:**
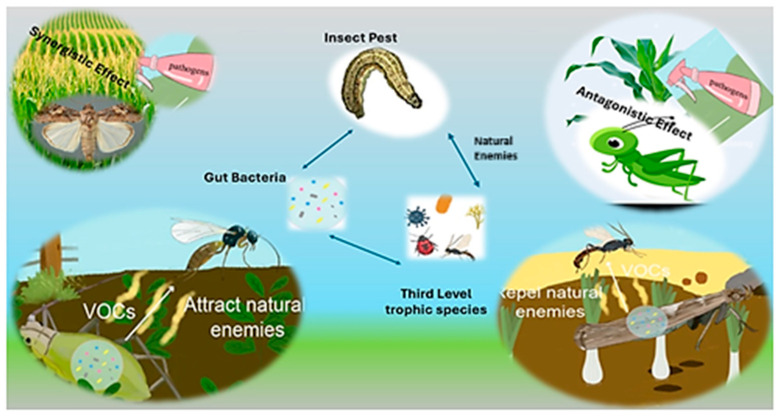
Gut microbiota impact plant–insect associations and third trophic-level organisms through either synergistic or antagonistic interactions with pathogens, and by the exhalation of volatile organic compounds (VOCs) that can attract or repel natural enemies. These mechanisms showcase the diversity of ecological roles of gut bacteria in insect-mediated interactions.

Insect–plant relationships may be mediated over various trophic levels by other organisms. This influence could take the form of plant volatile aromas (HIPVs), intentional alteration of the other’s physiology, or other, as yet undetermined processes. So far, the enhanced presence of organisms from higher trophic levels has been demonstrated to profoundly change the direct form of interactions between insects and plants (Figure 5).

For instance, gut bacteria of the targeted insects could have effects on the virulence of the microbial insecticides if pathogenic microorganisms were chosen to control insect pests. These bacteria from the gut can either activate or deactivate the chemicals. Moreover, gut microbiota composition can affect the production of particular plant volatiles (HIPVs) that induce behavioral responses in insect’s natural enemies. The outcomes of these interactions can have strong effects on the nature of insect–plant relationships and may add to the fabric of ecological interactions in an ecosystem.

## 10. Gut Bacteria Modulate the Efficacy of Microbial Insecticides

The evolutionary arms race of insects and their counter-pathogenic factors and toxins of microbial organisms leads to the acceleration of insect evolution, and thus produces various microbial insecticides such as entomopathogenic bacteria, fungi, and viruses [118]. Such agents have served as natural and good insect controllers. However, the virulence of these microorganisms is regulated by a number of biotic factors, and the gut microbiota of the insect host is an important barrier for infection or reproduction in either a permissive or non-permissive way.

For example, the cuticle of the bark beetle (*Dendroctonus valens*) infected by the entomopathogenic fungus *Beauveria bassiana* caused dysbiosis in the gut microbiota, resulting in overproliferation of *Erwinia* sp. and the increased mortality of beetles [119]. In diamondback moth (*Plutella xylostella*), ref. [36] found that Bt (*Bacillus thuringiensis*)-producing the *Cry1Ac* protoxin caused arousal in the midgut microbiota of the neonate larvae, and additionally, sometimes the *Bt* toxins interacted with the gut microbiota to kill the larvae. Conversely, the loss of the gut microbiota significantly decreased the susceptibility of *P. xylostella* larvae to *Bt* toxins [120]. Such synergistic relationships between pathogens and gut microflora have also been found in the European gypsy moth (*Lymantria dispar asiatica*) and the tobacco hornworm (*Manduca sexta*) [25,80].

Besides these synergistic effects, significant evidence of antagonistic interaction between pathogens and insect gut bacteria also exists. For instance, silkworm (*Bombyx mori*) challenged with the *Nosema bombycis* infection could increase the abundance of Enterococcus in the gut, and the *Enterococcus faecalis* LX10 was found to inhibit the spore load and infection rate of *Nosema bombycis* both in vitro and in vivo [121]. Enterococcus also suppressed the mycelial growth of entomopathogenic fungi in the experiments with the spruce beetle (*Dendroctonus rufipennis*) [122]. Intestinal antigens of locusts (*Schistocerca gregaria*) and scarab beetles (*Holotrichia oblita*, *Holotrichia parallela*, and *Anomala corpulenta*) also show similar antimicrobial activity [123,124].

It is notable that cooperation and inhibition may not be the preferred mode of interaction between pathogenic bacteria and gut bacteria. Caution is required, however; both the specific pathogen and host insect can strongly affect the outcome of these interactions, even within the same bacterial species (e.g., *Enterococcus faecalis*). Thus, in order to develop more specific and efficient MIAs, it is important to explore the complex tripartite interactions among pathogens, insect hosts, and their gut microbes.

## 11. Gut Bacteria Produce VOCs to Attract or Repel Natural Enemies of the Host

Entomopathogens are not the only trophic level important to insect–plant interactions; insects also have natural enemies that can influence and alter these interactions on a large scale [124]. In particular, predatory and parasitic species that attack herbivorous insects depend significantly upon volatile chemical signals to find their concealed prey in three-dimensional, structurally complex habitats [125]. Such cues are usually mediated by either specific host volatiles or other plant volatiles whose role can be attractive or repulsive to natural enemies. Recent work shows that volatile chemicals that come from insect gut bacteria can also affect behavior in their natural enemies. As an example, volatile chemotactic factors produced by bacteria in the honeydew of aphids (*Acyrthosiphon pisum*) have been shown to attract predators, and some compounds from *Staphylococcus sciuri* have been identified as attractants and oviposition stimulants for the predatory hoverfly (*Episyrphus balteatus*) [126]. In contrast, other research shows an inverse correlation of honeydew-associated bacteria with the attraction of the aphid parasitoid *Aphidius colemani* [127]. Additionally, ref. [127] showed that volatiles released by bacteria living in the frass of leek moth larvae (*Acrolepiopsis assectella*) act as olfactory signals for the parasitoid *Diadromus pulchellus* to find its host [128].

Gut bacteria can serve as repositories and are deposited into honeydew, frass, or other excretions from the insects while feeding and metabolic processes occur. The bacteria modulating the behavior of natural enemies are frequently related to insect-associated gut microbiota and can accordingly be divided into this category. Moreover, as mentioned earlier, gut bacteria release volatile chemicals that influence insect behaviors, including feeding and oviposition. Throughout evolution, natural enemies may have evolved the capacity to intercept and interpret these bacterial “chemical messages” to find their prey. This poses important questions regarding how insects might optimize their gut microbiota to limit the exploitation of these chemical signals by natural enemies.

**Figure 6:**
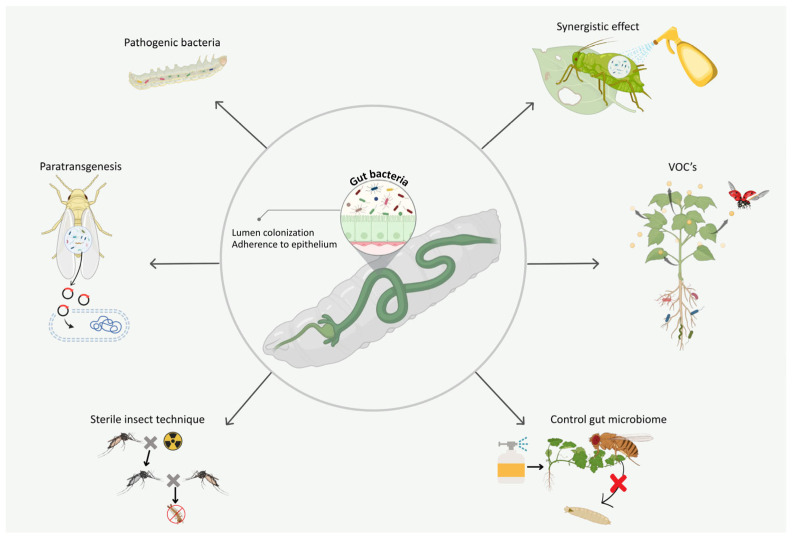
Gut microbiotas hold significant potential as pest management agents by influencing the biology of insect gut bacteria in insect–plant interactions. They modulate pathogens by either enhancing or inhibiting pest control agents and interact with natural enemies of host pests through the release of volatile organic compounds (VOCs). This figure depicts how gut microbiotas impact pest dynamics, influencing plant–pest relationships, pathogen control, and interactions with natural predators, ultimately contributing to sustainable pest management strategies.

Entomopathogenic bacteria and toxins have been developed and proven to be quite effective against a wide range of species for pest control. However, there is variation in the susceptibility of pests to the microbial insecticides, and some pests develop resistance to pathogenic bacteria [129,130,131]. This has underscored the need for more effective insecticidal bacteria. This has spawned much interest in gut bacteria as new sources of insecticidal agents (Figure 4 and Table 4). In addition, advances in molecular biology have expanded microbial control technologies beyond single bacteria. RNA interference (RNAi), sterile insect techniques (SIT), and paratransgenesis action mechanisms have also been combined with gut bacteria to improve pest control efficacy (Figure 6 and Table 4).

## 12. The Dual Insecticidal Power of Gut Bacteria: Direct Impacts and Untapped Potential

Certain gut bacteria have the potential to turn virulent under certain physiological or environmental conditions, or during an imbalance of the normal flora [132,133,134]. These pathogens are responsible for insect death in two basic ways: by starvation mediated by the production of gut toxins, and via sepsis associated with microbial dysbiosis [135]. An example is Enterobacter cloacae, which causes pathogenicity in cotton leafworm (*Spodoptera litura*) while feeding on insects through nutrient deprivation and disturbance in the native gut bacterial population [80]. In the midgut of *Manduca sexta* larvae, however, it is nonpathogenic, while artificial inoculation in the hemolymph causes a fast course to death [97]. The insect gut also contains *Serratia marcescens*, *Bacillus licheniformis*, *Pseudomonas aerugenosa*, *Proteus vulgaris*, *Alcaligenes faecalis*, and *Planococcus* spp. [136]. These microbes have potential to be used as bio-control agents as they possess pest-killing ability.

Sterile Insect Technology (SIT) consists of the large-scale rearing and gamma-irradiation of males, which are subsequently released into an area to be targeted, where they compete with wild males for wild female mating. This method has proven to be successful in pest control in *Tephritidae* spp. [137,138,139]. Nevertheless, it has been observed that sterile *C. capitata* males have greatly diminished mating competitiveness under field conditions. This decrease is correlated with a significant decrease in gut Klebsiella and an increase in pathogenic Pseudomonas [140]. Notably, the addition of *Klebsiella oxytoca* to these males can increase their mating competitiveness [141]. We argue that manipulation of gut microbiota, by promoting either the presence of Klebsiella or reducing Pseudomonas, can improve the competitive ability of sterile males to increase SIT effectiveness as a pest control tool.

Furthermore, besides the direct consequences on the fitness of the insect host itself, gut bacteria may modulate the dynamics of pests through their effects on insect–pathogen interactions that interfere with adaptive processes [140,142,143]. The gut microbiome makes an attractive target for pest control because interventions can be tailored to promote or reduce insect fitness. Moreover, plant VDP (Vegetative Defense Pathways)-restricted expression of antimicrobial peptides has been used to manipulate the plant-associated microbiota [144]. This provides a new cycle for the administration of herbivorous insect microbiota, where plants can either be engineered to produce antimicrobial agents or select for transmission of beneficial microbes, depending on pest control needs. Nevertheless, it is still required to further investigate the applicability and safety of the above methods.

In addition to affecting insect fitness, the gut bacteria can regulate the production of plant VOCs, which can also attract pests or their natural enemies [90,145,146]. Gut-derived VOCs and their potential to be utilized in pest management are somewhat unexplored, but with some promising records. One such example is the multi-component pheromone verbenone, derived from volatile pinene in the oleoresin of *Pinus contorta* [147,148], and has been reported to repel *Dendroctonus valens*. Although it was first described as a plant compound, its production in the gut by gut bacteria has only more recently been appreciated. We suggest that the inclusion of gut-derived VOCs in IPM programs might have at least two major purposes: to discover novel VOCs for pest control and to exploit gut bacteria of insects as “fermentation factories” responsible for VOC production as a microbial metabolism.

Moreover, Symbiont-Mediated RNA Interference (SMR) offers tremendous promise as a pest management technique. This approach includes encoding gut symbionts with double-stranded RNA (dsRNA), with the subsequent expression and secretion of dsRNA into the insect pest, and has been effective in diverse pest species [149,150,151]. For instance, in *Rhodnius prolixus*, *R. rhodnii* symbionts were engineered to express dsRNA that targets antioxidant pathways, leading to a decrease in the insect’s oviposition rate [152]. The same technique has been implemented in honeybees, proving the possibility of modifying gut microbiota for pest control purposes [153].

These are exciting new avenues for pest control that exploit a fine balance of pathogens, insect hosts, and gut bacteria. Improved knowledge of these microbial ecosystems is enabling more targeted, efficacious, and environmentally sound methods of pest management to be developed. Table 5 shows a detailed analysis of the microbial communities associated with the larval microbiomes across a wide range of insect families. It underscores the intricate and diverse microbial associations, revealing both shared and family-specific microbial profiles. This table not only highlights the ecological significance of these microbiomes but also provides valuable insights into the symbiotic relationships that contribute to insect development and health, offering a holistic perspective on microbial diversity in the insect world.

## 13. Fall Armyworm

The fall armyworm (*S. frugiperda*) is a highly destructive polyphagous pest that poses a serious threat to a broad spectrum of crops. The gut microbiota of insects plays a pivotal role in regulating their biology, physiology, and adaptive capacity to various environmental conditions. However, the specific composition and functional implications of the gut microbiota in *S. frugiperda* larvae feeding on different host plants remain poorly understood. In this study, metagenomic sequencing was used to investigate the structure, functional characteristics, and antibiotic resistance genes (ARGs) of the gut microbiota in *S. frugiperda* larvae transitioning from an artificial diet to four distinct host plants—maize, sorghum, tomato, and pepper.

The findings revealed significant differences in the gut microbiota profiles of *S. frugiperda* larvae depending on the host plant. The dominant phylum across all samples was *Firmicutes*, with *Enterococcaceae* being the most abundant family and Enterococcus the prevalent genus. Notably, *Enterococcus casseliflavus* was consistently detected across all host plants, highlighting its potential role as a core member of the gut microbiota in *S. frugiperda*. Metabolic pathway analysis identified key processes related to carbohydrate and amino acid metabolism that were critical for the adaptation of *S. frugiperda* gut microbiota across different hosts. For instance, *S. frugiperda* larvae feeding on sorghum exhibited an enriched regulation of the peptide/nickel transport system, while larvae fed on pepper showed increased expression of genes involved in glycolysis/gluconeogenesis and starch and sucrose metabolism, such as the 6-phospho-glucosidase gene. Furthermore, we identified the 20 most abundant *ARGs* present in the gut microbiota of *S. frugiperda* larvae across different host plants, with the *vanRC* gene being notably more abundant in maize-fed larvae.

This metagenomic sequencing analysis provides valuable insights into the dynamic variations in both the composition and functional attributes of the gut microbiota in *S. frugiperda* larvae depending on the host plant. These results underscore the intricate and evolving relationship between the host and its gut microbiota, highlighting the long-term effects of host plant transitions on microbial communities. This understanding may lead to more effective and targeted pest management strategies [19].

*Spodoptera frugiperda* represents one of the most significant agricultural threats globally, with documented damage to maize, wheat, and rice in various regions of China. As the need to understand the mechanisms driving global adaptation in *S. frugiperda* to diverse host plants intensifies, it becomes crucial to develop efficient and sustainable control strategies. In this study, two-sex life tables and 16S rDNA sequencing were used to assess both host fitness and gut microbial diversity in *S. frugiperda* larvae feeding on four different food sources. Life history parameters such as pupa weight and nutrient utilization indices revealed that host fitness ranked from highest to lowest as follows: artificial diet > maize > wheat > rice. Our analysis also showed that the composition and diversity of the gut microbiota were significantly influenced by the type of food consumed, with low-abundant bacteria being the primary drivers of microbial diversity. Notably, maize-fed larvae exhibited the most diverse gut microbiota. Functional analysis of gut microbes that displayed significant differences in abundance revealed a marked enrichment in pathways related to nutrient and vitamin metabolism, which are crucial for host adaptation. Additionally, five genera (*Acinetobacter*, *Variovorax*, *Pseudomonas*, *Bacillus*, and *Serratia*) were positively correlated with host fitness, and one genus (Rahnella) was negatively correlated with host fitness. These findings offer new perspectives on the factors influencing the successful adaptation of *S. frugiperda* to diverse hosts and provide deeper insights into the complexities of microbiota–host interactions, which are essential for the development of more effective pest management strategies [27]. This study highlights the importance of the gut microbiome in shaping the fitness and adaptability of *S. frugiperda*, offering a novel approach for future pest management strategies that target microbiota–host interactions.

## 14. Conclusions

The present study has shown that gut microbiota is an ecological driver in insects, including plant interactions. Insect–bacteria gut associations can affect host insect adaptability and fitness, host plant selectivity, and host plant growth and defense against modified organisms, pathogenic microorganism virulence, and attraction or repulsion of insects to natural enemies. These complex relationships are indicative of the centrality of the gut microbiota as a key component for new and sustainable strategies for pest control. The use of gut bacteria in managing pests has expanded with the development of RNA interference (RNAi) and para-transgenesis, providing an eco-friendly and sustainable alternative to conventional chemical pesticides. As a promising alternative against the environmental issues caused by traditional pest management, biocontrol agents, especially those acting on insect gut microbiota, have shown their superiority.

Nevertheless, the application of gut bacteria for biocontrol is associated with few practical constraints despite their potential. We do not fully know the mechanisms through which insects, microbes, and plants interact, especially because these interactions are so complex, and axenic insects are difficult to produce. Although axenic rearing methods have been useful to dissect and reconstruct these relations, they are limited to a narrow set of pest species. In addition, most gut bacteria are unculturable, making it difficult to investigate their function and potential applications. While improvements in culturomics and large-scale culturing techniques have been very encouraging, the issues of growing and releasing such bacteria remain challenging.

A further complication is the fact that insect–plant–microbe interactions can be variable in nature (e.g., abiotic variables, such as climate change) when these factors may influence the outcome. Laboratory studies, however, only rarely mimic the real-life dynamics of these interactions. Thus, we support those future studies of insect–microbe–plant interactions should incorporate ecological variables (such as climate) so laboratory-based results can be readily translated to field scenarios.

Furthermore, efficiency, cost, and scalability also need to be considered when integrating biological control techniques like RNAi or Sterile Insect Technique (SIT) in conjunction with gut bacteria-based approaches. Before these approaches can be implemented on a broader scale, several factors will need to be addressed, including the technical limitations of dsRNA large-scale production and field application, and the overall expenses and logistical support of the SIT. However, the functional roles of gut bacteria in insect–plant interactions have only been accessible to direct investigation for the last few years with the use of new high-throughput techniques such as metagenomics, transcriptomics, and other related technologies, providing valuable foundational information for identification and design strategies toward novel pest control methods.

In addition, as regards the future, the study of the molecular bases that determine the exchanges between insects, plants, and the fauna of their guts also offers a large scientific and applied potential. In light of increasing resistance of pests to conventional control methods, exploration of pest-microbiota symbioses represents an appealing alternative approach toward new, environmentally friendly pest management approaches. Future research will need to continue clarifying and cataloguing the functional diversity of gut bacteria, consider the potential scale of strain-level variation, and develop increased synergy between ecological, molecular, and field-based research. Not only will these types of research help to build our understanding of insect ecology, they will also provide us with sustainable methods for pest control. Assessing microbiome-mediated biocontrol efficacy under climate variability while evaluating the effectiveness of microbiome-mediated biocontrol strategies in the face of climate variability, researchers must conduct comprehensive studies that account for diverse environmental conditions. These investigations should include field trials across different geographical regions and seasons, as well as controlled experiments simulating various climate scenarios. Factors such as temperature fluctuations, precipitation patterns, and extreme weather events should be considered to determine their impact on microbial communities and their biocontrol capabilities. Additionally, long-term monitoring of microbial population dynamics and their interactions with target pests or pathogens is crucial for understanding the resilience and adaptability of biocontrol agents under changing climatic conditions. By integrating data from these multifaceted approaches, scientists can develop more robust and climate-resilient biocontrol strategies, ultimately enhancing sustainable agricultural practices in the face of global climate change.

Addressing technological limitations is imperative, particularly in enhancing dsRNA delivery through engineered symbionts such as *Serratia* spp. This process entails modifying Serratia strains to augment their ability to produce and release dsRNA. Furthermore, optimizing the dsRNA sequence length and structure is critical to ensure its stability and efficacy. It is also necessary to develop methods for the large-scale, cost-effective production of dsRNA within Serratia. Incorporating genes that enhance colonization and persistence in target insects is of significant importance. Additionally, engineering Serratia to express dsRNA-binding proteins can improve uptake. Another area of focus is the development of nanoparticle-based delivery systems that are compatible with Serratia. Investigating the factors influencing Serratia colonization in target insects is crucial, as is optimizing the establishment and persistence of symbionts within insect gut environments.

In summary, despite remaining obstacles, the obstacle of pest control turns out to be the exploitation of insect gut bacteria itself. Through increased understanding of these complex symbiotic interactions and development in the technical aspects needed to exploit them, we will be able to cater for an improved, more effective, sustainable, and eco-friendly pest control in the future, and make it feasible to respond to the expanding problem of the development of pest resistance.

## Figures and Tables

**Table 1:** Relation of microbial families to lepidopteran families along with various species.

Microbial Family	Lepidoptera Family and Species	References
Bacteroidetes	Noctuidae (*Spodoptera frugiperda*), Pieridae (*Pieris canidia*)	[19,23,24]
Firmicutes	Noctuidae (*Spodoptera litura*), Lymantriidae (*Lymantria dispar*)	[4,6,25]
Proteobacteria	Saturniidae (*Bombyx mori*), Pieridae (*Pieris rapae*)	[3,20]
Actinobacteria	Pieridae (*Danaus plexippus*), Noctuidae (*Plutella xylostella*)	[3,13]
Ascomycota	Saturniidae (*Bombyx mori*), Tortricidae (*Cydia pomonella*)	[4,6]
Basidiomycota	Noctuidae (*Spodoptera frugiperda*), Erebidae (*Lonomia obliqua*)	[23,26]
Entomophthorales	Saturniidae (*Bombyx mori*), Sphingidae (*Hylesia metabus*)	[19,25]
Zygomycota	Noctuidae (*Helicoverpa armigera*), Saturniidae (*Actias luna*)	[19,24]
Archaea (Methanogens)	Noctuidae (*Spodoptera frugiperda*), Pieridae (*Pieris canidia*)	[27]
Fusobacteria	Lymantriidae (*Lymantria dispar*), Noctuidae (*Spodoptera litura*)	[19,28]
Euglenozoa	Pieridae (*Danaus plexippus*), Noctuidae (*Plutella xylostella*)	[13,29]
Cercozoa	Noctuidae (*Spodoptera frugiperda*), Tortricidae (*Cydia pomonella*)	[3,29]
Glomeromycota	Saturniidae (*Bombyx mori*), Lymantriidae (*Lymantria dispar*)	[30,31]
Chytridiomycota	Pieridae (*Pieris rapae*), Saturniidae (*Actias luna*)	[32]
Chlorophyta	Noctuidae (*Helicoverpa armigera*), Tortricidae (*Cydia pomonella*)	[24]
Malawimonadida (Protists)	Noctuidae (*Spodoptera frugiperda*), Saturniidae (*Bombyx mori*)	[19,27]
Stramenopiles	Lymantriidae (*Lymantria dispar*), Noctuidae (*Spodoptera litura*)	[28]

**Table 2:** Factors influencing gut microbiota composition across host, environmental, and microbial dimensions.

Category	Factor	Effect on Gut Microbiota
Host-related Factors	Diet Composition	The types of food and nutrients available to the host influence microbial communities.
Host-related Factors	Developmental Stage	Microbial composition varies across different life stages (e.g., larvae, pupae, adults).
Host-related Factors	Immune System	The host immune response can regulate microbial colonization and diversity.
Environmental Factors	Habitat	The physical environment, such as vegetation type, affects microbial diversity.
Environmental Factors	Temperature	Temperature can influence microbial growth and community structure.
Environmental Factors	Humidity	Humidity levels can affect the survival and activity of gut microbes.
Microbial Interactions	Symbiotic Relationships	Beneficial microbes in mutualistic relationships can shape gut microbiota.
Microbial Interactions	Antagonism	Antagonistic interactions between microbes can affect microbial diversity and function.

**Table 3:** Relative abundance of bacterial classes in the gut microbiomes of Lepidoptera species derived from these references or calculated based on these sources. The columns include the bacterial phylum (Phylum), the specific bacterial class (Class), average relative abundance (Mean), and standard deviation of the relative abundance (SD). These data provide insights into the diversity and distribution of bacterial communities within Lepidoptera gut ecosystems.

Phylum	Class	Mean	SD
Bacteroidota	Bacteroidia	0.085900266	0.084621476
Proteobacteria	Alphaproteobacteria	0.253684521	0.196852416
Proteobacteria	Gammaproteobacteria	0.315196589	0.226446002
Firmicutes	Bacilli	0.162957572	0.179644224
Actinobacteriota	Actinobacteria	0.072742687	0.100153701
Actinobacteriota	Thermoleophilia	0.00494136	0.015191977
Firmicutes	Clostridia	0.056172865	0.145360754
Verrucomicrobiota	Verrucomicrobiae	0.005398711	0.015210373
Firmicutes	Negativicutes	0.001961527	0.005447803
Planctomycetota	Planctomycetes	0.004907338	0.013982851
Cyanobacteria	Cyanobacteriia	0.002196259	0.009989121
Acidobacteriota	Acidobacteriae	0.003262416	0.008807503
Abditibacteriota	Abditibacteria	0.001797995	0.009358712
Fusobacteriota	Fusobacteriia	0.002613247	0.007239766
Actinobacteriota	Acidimicrobiia	0.001258584	0.005626321
Myxococcota	Polyangia	0.001640708	0.005979046
Verrucomicrobiota	Chlamydiae	0.001354805	0.015833571
Bdellovibrionota	Oligoflexia	0.001410108	0.009619666
Unknown	Unknown	0.001346226	0.006998769
Acidobacteriota	Blastocatellia	0.001360653	0.004650909

**Table 4:** Impact of the gut bacteria on the insect–plant interaction. This table provides a summary of how gut bacteria mediate different aspects of insect–plant interactions, with the specific bacteria that play a role in digestion, defense, and plant–microbe interactions.

Insect	Order	Gut Bacteria	Function of Bacteria
*Cossus cossus*	Lepidoptera	*Bacillus circulans*	Production of cellulase
*Trichoplusia ni*	Lepidoptera	*Agrobacterium* sp.	Degradation of alkaloids
*Thitarodes xiaojinensis*	Lepidoptera	*Raoultella terrigena*	Degradation of quercetin
*Plutella xylostella*	Lepidoptera	*Midgut microbiota*	Assisting plant toxins to kill insects
*Helicoverpa zea*	Lepidoptera	*Serratia marcescens*	Killing insects in synergy with plant defense
*Spodoptera frugiperda*	Lepidoptera	*Enterococcus* sp.	Interacting with plant defenses to kill insects
*Spodoptera littoralis*	Lepidoptera	*Gut microbiota*	Dysbiosis of gut microbiota is detrimental to larval survival
*Diatraea saccharalis*	Lepidoptera	*Bacillus pumilus*	Production of cellulase
*Dendroctonus valens*	Coleoptera	*Gutmicrobiota*	Assisting beetles to determine the suitability of the pine tree for colonization
*Cyrtotrachelus buqueti*	Coleoptera	*Bacillusvelezensis*	Production of cellulase
*Holotrichia paralella*	Coleoptera	*Pseudomonas* sp.	Production of cellulase
*Osphranteria coerulescens*	Coleoptera	*Bacillussafensis*	Production of cellulase
*Cassida rubiginosa*	Coleoptera	*Stammera*	Production of pectinase
*Hypomeces squamosus*	Coleoptera	*Enterobacter hormaechei*	For feeding and females for egg laying
*Cassida rubiginosa*	Coleoptera	*Stammera*	Production of pectinase
*Hypomeces squamosus*	Coleoptera	*Enterobacter hormaechei*	For feeding and females for egg laying
*Sphenophorus levis*	Coleoptera	*Gut microbiota*	Production of pectinase
*Odontotaenius disjunctus*	Coleoptera	*Bacteroidetes* sp.	Biological nitrogen fixation
*Melolontha hippocastani*	Coleoptera	*Burkholderia* sp.	Recycling of nitrogen in larvae
*Hylobius abietis*	Coleoptera	*Enterobacteriaceae* sp.	Degradation of terpenoids
*Curculio chinensis*	Coleoptera	*Acinetobacter* sp.	Degradation of tea saponin
*Hypothenemus hampei*	Coleoptera	*Pseudomonas fulva*	Degradation of caffeine
*Dendroctonus valens*	Coleoptera	*Novosphingobium* sp.	Degradation of phenolic naringenin
*Psylliodes chrysocephala*	Coleoptera	*Pantoea* sp.	Degradation of isothiocyanates
*Acrobasis nuxvorella*	Coleoptera	*Bacillus pumilus*	Degradation of Carya illinoinensis tannins
*Macrotermes gilvus*	Isoptera	*Provedencia* sp.	Production of cellulase
*Cryptotermes brevis*	Isoptera	*Bacillus* sp.	Production of ligninase
*Odontotermes obesu*	Isoptera	*Trabulsiella* sp.	Production of ligninase
*Reticulitermes chinensis*	Soptera	*Enterobacter hormaechei*	Production of ligninase
*Bactrocera dorsalis*	Diptera	*Citrobacter* sp.	Attracting female flies to lay eggs on the host fruit by VOCs
*Ceratitis capitata*	Diptera	*Enterobacteriaceae* sp.	Biological nitrogen fixation
*Bactrocera dorsalis*	Diptera	*Morganella morganii*	Hydrolyzing nitrogenous waste and providing metabolizable nitrogen
*Riptortus pedestris*	Hemiptera	*Burkholderia* sp.	Supplementation of essential amino acids and B vitamins
*Dysdercus fasciatus*	Hemiptera	*Coriobacterium glomerans*	Supplementation of B vitamins
*Sirex noctilio*	Hymenoptera	*Streptomyces*	Production of cellulase
*Frankliniella occidentalis*	Thysanoptera	*Erwinia* sp.	Attracting larvae

**Table 5:** Larval microbiomes composition and associated microbiome communities across insect families.

Insect Family	Larval Microbiome	Microbiome Communities
Pieridae	*Enterococcus*, *Bacillus*, *Pseudomonas*	Enterococcus, Pseudomonas
Noctuidae	*Enterococcus*, *Serratia*, *Bacillus*	Enterococcus, Serratia
Saturniidae	*Enterococcus*, *Acinetobacter*	Enterococcus, Bacillus
Tortricidae	*Bacillus*, *Enterococcus*, *Pantoea*	Bacillus, Enterococcus, Pantoea
Lymantriidae	*Enterococcus*, *Bacillus*, *Lactobacillus*	Enterococcus, Bacillus, Lactobacillus
Nymphalidae	*Spiroplasma*, *Enterococcus*	Spiroplasma, Enterococcus
Hesperiidae	*Enterococcus*, *Bacillus*	Enterococcus, Bacillus
Papilionidae	*Bacillus*, *Enterococcus*	Bacillus, Enterococcus
Lycaenidae	*Pseudomonas*, *Enterococcus*, *Bacillus*	Pseudomonas, Enterococcus
Pierinae	*Bacillus*, *Enterococcus*	Bacillus, Enterococcus
Erebidae	*Acinetobacter*, *Serratia*	Acinetobacter, Serratia
Crambidae	*Bacillus*, *Enterococcus*	Bacillus, Enterococcus
Geometridae	*Bacillus*, *Enterococcus*	Bacillus, Enterococcus
Sphingidae	*Acinetobacter*, *Enterococcus*	Acinetobacter, Enterococcus
Cossidae	*Bacillus*, *Streptomyces*, *Pseudomonas*	Bacillus, Streptomyces, Pseudomonas
Arctiidae	*Enterococcus*, *Bacillus*, *Pseudomonas*	Enterococcus, Bacillus, Pseudomonas
Sattleridae	*Bacillus*, *Enterococcus*, *Pseudomonas*	Bacillus, Enterococcus, Pseudomonas
Pyralidae	*Enterococcus*, *Lactobacillus*	Enterococcus, Lactobacillus

## Data Availability

Not applicable.
